# Short-Term Evaluation of Left Ventricular Mass and Function in Children With Growth Hormone Deficiency After Replacement Treatment

**DOI:** 10.3389/fped.2018.00174

**Published:** 2018-06-19

**Authors:** E. Gómez-Guzmán, M. D. Cañete, R. Valle-Martos, R. Cañete, M. Valle, L. Jiménez-Reina, J. Caballero-Villarraso

**Affiliations:** ^1^Cardiology Unit, Hospital Reina Sofía de Córdoba, Cordova, Spain; ^2^Instituto Maimonides de Investigación Biomédica de Cordoba, Cordova, Spain; ^3^Hospital Universitario La Paz, Madrid, Spain; ^4^Clinical Analysis Services, Hospital Valle De Los Pedroches, Cordova, Spain; ^5^Facultad de Medicina y Enfermería, Universidad de Córdoba, Cordova, Spain

**Keywords:** growth hormone deficiency, prepubertal children, heart, GH replacement therapy, left ventricular mass

## Abstract

**Background:** Our study was designed to assess the effects of GHD on nutritional and metabolic parameters, brain natriuretic peptide (BNP) levels, and left ventricular mass (LVM) in prepubertal children and after short-term GH replacement therapy.

**Materials and Methods:** This prospective study enrolled 81 children. We compared 40 GHD children (16 males and 24 females) to 41 healthy children (control group) (18 males and 23 females). All subjects were at Tanner Stage I (aged 7–11 years). At the baseline, a blood sample was drawn and echocardiographic images were obtained. These tests were repeated on the GHD subjects after 6 months of GH replacement therapy. Body surface, weight, size, blood pressure, heart rate, glucose, insulin, HOMA-IR, HOMA-β, QUICKI, cholesterol, HDLc, LDLc, triglycerides, IGF1, and IGFBP3 were measured. Indexed LVM, diastolic and systolic diameter (dD-sD), diastolic and systolic LV function, isovolumic relaxation time, right ventricle function, and BNP levels were obtained through echocardiography. These parameters were correlated to growth factors. Data were analyzed using Student's *t*-test or U-Mann–Whitney-test and Pearson's correlation, considering *p* < 0.05 to be significant.

**Results:** Indexed LVM was smaller in GHD patients than in controls, whereas diastolic and systolic functions, BNP, metabolic, and nutritional profiles were similar. After treatment, nutritional and metabolic profiles significantly improved, though diastolic and systolic functions did not seem to have changed. There was a significant increase in LVM. Indexed LVM was similar to that of controls. Significant correlations were obtained between LVM-IGF1 and sD-IGFBP3.

**Conclusions:** GHD in childhood is associated with a lower indexed LVM. In the short-term, GH increases the indexed LVM, while maintaining normal systolic and diastolic functions, BNP, and an improved lipid profile.

## Introduction

Low secretion of growth hormone (GH) is accompanied by a series of auxologic, clinical, biochemical, and metabolic abnormalities which characterize growth hormone deficiency (GHD), and also influence GH-dependent growth factors (1). Adult patients with established GHD have an increased risk of cardiovascular disease and early mortality (2), and the condition has been associated with an unfavorable lipid profile, an increase in body fat, an increase in Homeostasis Model Assessment of Insulin Resistance index (HOMA-IR), early arteriosclerosis and a decrease in fibrinolytic activity (3–6), a reduction in left ventricular mass (LVM), a smaller idle and after-stress ejection fraction (EF), diastolic dysfunction, and an increase in brain natriuretic peptide (BNP) levels (7). Replacement therapy with GH has beneficial effects on these cardiovascular risk factors, improves ventricular function, and decreases BNP, thus suggesting that GH may be effective in reducing mortality from cardiovascular disease (8, 9). There are data indicating that teenagers who suffer severe GHD and who complete replacement therapy with GH experience more adverse events affecting body composition, lipid profile, bone mineral density, morphology and heart function, and fibrinolytic activity (6–10). There are fewer studies in prepubertal children suggesting that childhood GHD causes the same effects, although there are data showing a decrease in LVM. Similarly, there are a few studies assessing the short-term effects of GH action in these patients, which also include BNP as a heart function marker.

The objective of the present study is to assess the influence of GHD on heart function, LVM, BNP, and lipid profiles compared to a control population, as well as the effects of replacement therapy with GH in a pediatric population without pubertal development after 6 months of treatment.

## Materials and methods

This was a prospective, observational, case-control study in prepubertal GHD children of both sexes. This study with consecutive sampling was carried out jointly at Pediatric Cardiology and Endocrinology Sections, Reina Sofía University Hospital of Córdoba (RSUH), and the Clinical Analysis Service (CAS) at RSUH. The study was carried out in accordance with the recommendations of the RSUH Ethics and Research Committee. Follow-up of children without GHD (longitudinal study) was not authorized. In all cases, a written informed consent was obtained from a parent or legal guardian in accordance with the Helsinki Declaration. The protocol was approved by the RSHU Committee, and in no case did any participant, parent, or guardian receive any financial compensation for participating.

### Cross-sectional study

This study enrolled 81 children. We included 40 GHD children (16 males and 24 females) and 41 healthy children (control group) (18 males and 23 females). Physical examinations by pediatric endocrinologists at the beginning and again at the end of the study established that the children were all Tanner Stage I (prepubertal). They were all between 7 and 10 years of age.

The diagnoses of GHD children were made according to Growth Hormone Research Society criteria with a maximum GH peak of 7.3 ng/mL in the two GH provocation tests, measured by means of a monoclonal assay. Other causes of short stature were excluded (11).

Exclusion criteria were as follows: children with known congenital infections, genetic abnormalities and congenital malformations or syndromes with an unknown genetic defect, concomitant metabolic disorder or general disease, and failure to meet any of the above criteria.

### Longitudinal study

GHD subjects started GH therapy at 0.030 mg/Kg per day for 6 months. None of the subjects showed signs of puberty during the study period. Changes in anthropometric parameters, biochemical analysis, and heart function were monitored for this group in two visits before the initiation of the GH therapy and 6 months after.

### Baseline characteristics and biochemical analysis

At the first visit, a clinical history was obtained, anthropometric parameters such as body weight (Kg), height (cm), body surface area (cm^2^), systolic and diastolic blood pressure (mmHg), and heart rate (beats/min) were determined via standardized procedures.

Coincident with routine clinical testing, 3 ml venous blood samples were collected with minimal bodily injury from each subject after 12 h of fasting between 08:30 and 9:30 a.m. Samples were drawn into Vacutainer® SST (BD) tubes, allowed to clot in the cold for 30 min., and later centrifuged at 1,500 × g for 10 min. at 4°C. The samples were analyzed in the CAS at RSUH.

Serum glucose concentrations were measured using the hexokinase method; total cholesterol (total-c), high-density lipoprotein cholesterol (HDLc) and triglycerides (TG) were analyzed via oxidation-peroxidation. Low-density lipoprotein cholesterol (LDLc) was calculated by means of Fredewald's formula (when triglycerides were above 300 mg/dL), as: [LDLc = Total Cholesterol − (HDLc + TG/5)]. Insulin and BNP concentrations were quantified via chemiluminescent microparticle immunoassay. IGFBP-3 and IGF-1 were determined by immunoradiometric analysis using a Packard-Cobra II model 5002 solid scintillation counter.

The Homeostasis Model Assessment for Insulin Resistance (HOMA-IR), HOMA-β index, and the Quantitative Insulin Sensitivity Check Index (QUICKI) were calculated using the fasting plasma glucose and insulin values following the formulas: HOMA-IR = fasting glucose (mmol/L) × fasting insulin (μU/mL)/22.5; HOMA-β index = 20x fasting insulin (μU/mL)/(fasting glucose (mmol/L)-3.5 and QUICKI = 1/(log fasting insulin (μU/mL)+log fasting glucose (mg/dL).

### Heart study

Patients and controls underwent a standard electrocardiogram. Frequency, rhythm, cardiac axis, and signs of ventricular hypertrophy or repolarization disorders were analyzed. M-mode, two-dimensional, and pulsed doppler echocardiographic studies were performed with an echocardiograph (Philips HD11 with S4 multifrequency probe with harmonics), according to the recommendations of the American Society of Echocardiography (12).

The following measures were obtained: telediastolic and telesystolic LV diameter (dDLV, sDLV), posterior wall thickness in diastole (PWTD) and intraventricular septum thickness in diastole (IVSTD). Using these parameters, the ejection fraction (EF) and the LVM were calculated: EF = (dDLV^3^ − sDLV^3^/dDLV^3^) × 100; LVM = 0.8 × (1.04 × (LVIDD+IVSTD+PWTD)^3^ − LVIDD^3^) + 0.6 g.

We also obtained Isovolumic relaxation time (IVRT), the E/A wave ratio (E, passive filling of the LV/A, atrial filling), the E/E′ ratio (E′, movement of the LV wall through tissue doppler) and the tricuspid annular plane systolic excursion (TAPSE).

### Statistical analysis

Statistical analysis was performed using SPSS software version 22 for Windows (SPSS Inc., Chicago IL, USA). Abnormal values (outliers) were excluded. Results were expressed as mean ± *SD* with a 95% confidence interval (CI). The distribution of each variable was tested for departure from Gaussian distribution, and variance equality was controlled by the Snedecor *F*-test. Mean values for the groups were compared using Student's *t*-Test for paired observations (GHD baseline group vs. GHD after 6 months of treatment) as well as for unpaired observations (control group vs. GHD baseline group, and control group vs. GHD after 6 months of treatment). In the longitudinal study, correlations between changes in variables were evaluated using Pearson's correlation coefficient and regression analysis.

## Results

### Baseline characteristics and biochemical analysis

Clinical and anthropometric details were recorded for both groups (control and GHD) at baseline, that is, before treatment was given to the GHD group (Table 1). Weight, height, and body surface were significantly lower, as expected, in GHD children than in controls. After 6 months of GH replacement therapy, weight (30.36 ± 7.81), height (131.88 ± 10.9), and body surface (1.05 ± 0.17) were measured and compared to the same groups values before treatment had been started, and, as expected, there were differences (*p* = 0.000, respectively).

**Table 1:** Baseline characteristics of participants.

	**Control (*n*:41)**	**GHD baseline (*n*:40)**	***P***
Weight (Kg)	33.92 ± 9.60	28.13 ± 7.41	< 0.005
Height (cm)	137.24 ± 10.58	127.28 ± 11.09	< 0.001
Body surface (cm^2^)	1.13 ± 0.19	0.99 ± 0.17	< 0.001
Systolic blood pressure (mmHg)	102.17 ± 17.56	103.35 ± 10.57	0.766
Diastolic blood pressure (mmHg)	65.44 ± 8.80	62.95 ± 9.17	0.262
Heart rate (beats/min)	79.32 ± 9.86	83.43 ± 15.21	0.526

*Values are mean ± SD*.

Biochemical analysis in controls and GH patients at baseline and after GH treatment are shown in Table 2. Column A shows the results of the cross-sectional study, and column B the longitudinal study.

**Table 2:** Biochemical parameter changes over 6 months of GH therapy in patients with GHD compared with controls.

	**A.1 Control (*n*:41)**	**A.2 GHD baseline (*n*:40)**	***p*-value**	**B.1 GHD baseline (*n*:40)**	**B.2 GHD 6 months (*n*:40)**	***p*-value**
Glucose (mmol/L)	4.74 ± 0.33	4.72 ± 0.46	0.860	4.72 ± 0.46	4.94 ± 0.45	0.011
Total-c (mg/dL)	161.7 ± 26.37	175.4 ± 29.23	0.029	175.4 ± 29.23	175.6 ± 31.27	0.694
HDL-c (mg/dL)	50.41 ± 10.46	54.38 ± 13.01	0.135	54.38 ± 13.01	59.18 ± 13.75	0.035
LDL-c (mg/dL)	99.90 ± 24.60	109.1 ± 21.67	0.036	109.1 ± 21.67	99.82 ± 29.09	0.006
TG (mg/dL)	54.92 ± 26.05	58.28 ± 26.44	0.620	58.28 ± 26.44	66.15 ± 23.58	0.090
Insulin (μU/mL)	7.34 ± 1.63	7.33 ± 1.72	0.993	7.33 ± 1.72	8.25 ± 1.67	0.000
HOMA-IR	1.55 ± 0.35	1.53 ± 0.36	0.800	1.53 ± 0.36	1.80 ± 0.37	0.000
Quicki-index	0.66 ± 0.04	0.36 ± 0.01	0.000	0.36 ± 0.01	0.63 ± 0.03	0.000
HOMA β index	124.1 ± 46.1	126.1 ± 51.98	0.877	126.1 ± 51.98	132.1 ± 73.17	0.748
IGFBP3 (μg/mL)	2.57 ± 1.01	2.44 ± 0.72	0.607	2.44 ± 0.72	2.74 ± 0.60	0.010
IGF1 (ng/mL)	200.3 ± 94.04	185.2 ± 198.5	0.322	185.2 ± 198.5	297.3 ± 129.1	0.000

*HOMA-IR, Homeostasis Model Assessment-Insulin Resistance; Quicki-index, Quantitative Insulin Sensitivity Check Index; IGFBP3, Insulin-like Growth Factor Binding Protein 3; IGF1, Insulin-like growth factor. All values are expressed as mean ± SD*.

Total cholesterol and LDLc levels were significantly higher and QUICKI was lower in GHD patients at baseline than in the control group, although within normal values for age. There were no significant differences at baseline with respect to the rest of the biochemical parameters studied (Table 2 A1 vs. A2). In the longitudinal study, LDLc levels were significantly lower, and glucose, HDLc, Insulin, Homa-IR, QUICKI and growth factors IGF1, IGFBP3 increased significantly after 6 months of GH replacement therapy in these children compared to GHD patients at baseline (Table 2 B1 vs. B2). Finally, after comparing the GHD children after 6 months of replacement therapy to the control group, all parameters remained significantly elevated except LDLc, approaching baseline values; however, values were always within the normal range for age.

### Heart study

**Figure 1:**
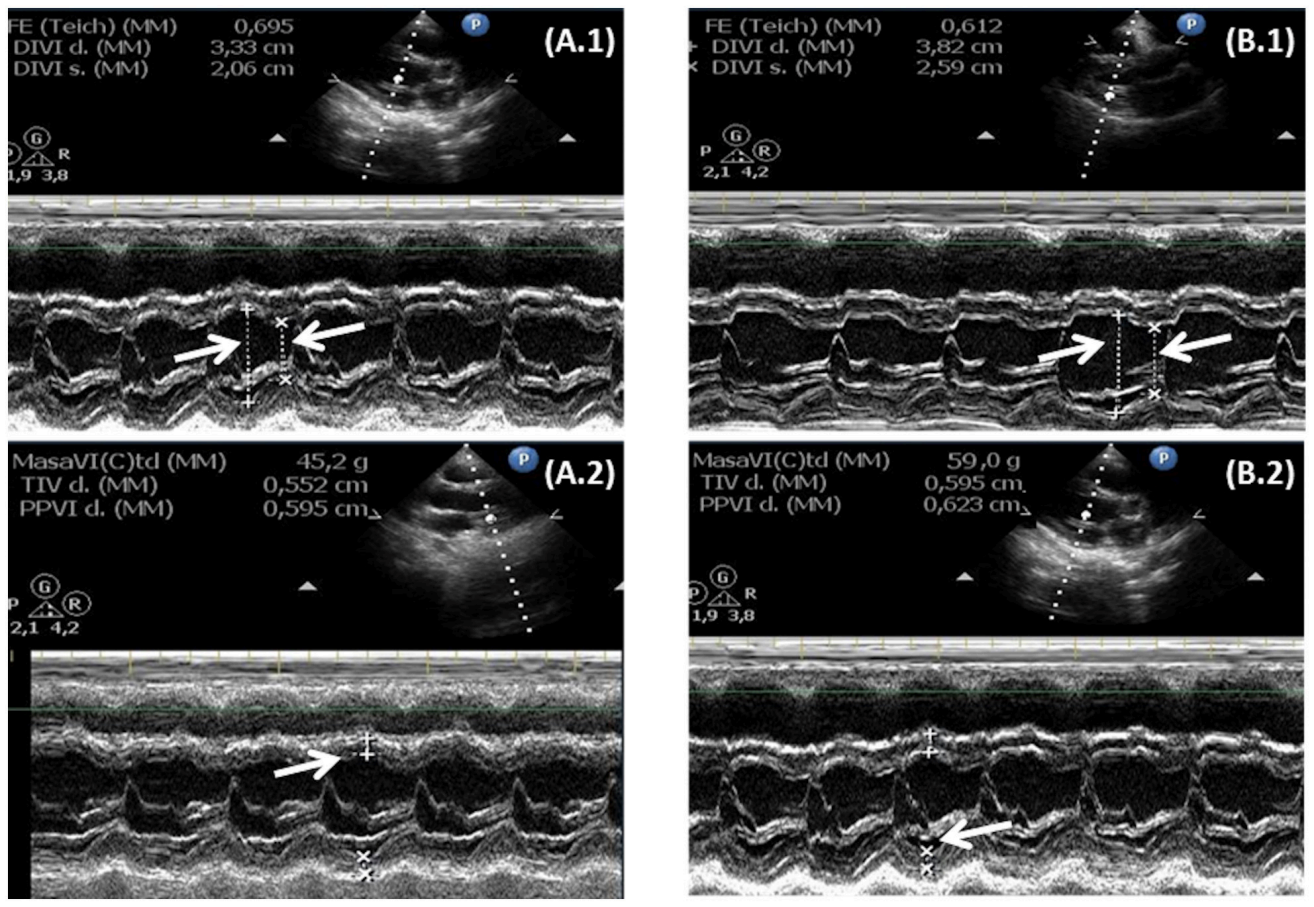
Image and data of left ventricular function and mass index (LVMI) measured by echocardiography mode M in GHD infants at baseline **(A.1,A.2)** and after 6 months with GH replacement therapy **(B.1,B.2)**. Arrows indicate the systolic and diastolic diameter.

Data and measurements for LV function and mass by echocardiography before and 6 months after GH replacement therapy are shown in Figure 1.

Table 3 shows the results obtained in terms of size, function and mass. BNP levels and heart weight from echocardiographic measurements performed on all patients included in the study. Column A shows the results of the cross-sectional study, column B the longitudinal study and Colum C shows the results observed when we compared GHD children after 6 months with recombinant GH vs. control group.

**Table 3:** Echocardiographic data of GH deficiency and normal growth infants.

	**A.1 Control (*n*:41)**	**A.2 GHD baseline (*n*:40)**	***p*-value**	**B.1 GHD baseline (*n*:40)**	**B.2 GHD 6 months (*n*:40)**	***p*-value**	**C.1 GHD 6 months (*n*:40)**	**C.2 Control (*n*:41)**	***p*-value**
**SIZE**									
Systolic diameter of LV (mm)	25.93 ± 3.50	23.62 ± 3.40	0.003	23.62 ± 3.40	23.63 ± 2.56	0.976	23.63 ± 2.56	25.93 ± 3.50	0.001
Diastolic diameter of LV (mm)	39.49 ± 3.80	37.17 ± 3.60	0.006	37.17 ± 3.60	38.41 ± 3.89	0.007	38.41 ± 3.89	39.49 ± 3.80	0.208
**FUNCTION**									
EF (%)	64.30 ± 5.59	67.06 ± 5.93	0.055	67.06 ± 5.93	66.83 ± 5.34	0.850	66.83 ± 5.34	64.30 ± 5.59	0.040
E/A relation	1.93 ± 0.52	1.89 ± 0.51	0.741	1.89 ± 0.51	1.87 ± 0.46	0.800	1.87 ± 0.46	1.93 ± 0.52	0.583
E/E'relation	6.96 ± 1.64	6.54 ± 1.83	0.052	6.54 ± 01.83	6.84 ± 2.56	0.502	6.84 ± 2.56	6.96 ± 1.64	0.390
IVRT (mseg)	87.66 ± 21.10	79.51 ± 16.48	0.062	79.51 ± 16.48	77.08 ± 14.38	0.457	77.08 ± 14.38	87.66 ± 21.1	0.010
TAPSE (mm)	22.04 ± 4.03	21.03 ± 2.77	0.193	21.03 ± 02.77	21.75 ± 2.92	0.137	21.75 ± 2.92	22.04 ± 4.03	0.713
BNP (pg/mL)	17.54 ± 15.09	20.58 ± 13.88	0.231	20.58 ± 13.88	18.85 ± 17.84	0.573	18.85 ± 17.84	17.54 ± 15.09	0.874
**MASS**									
LV mass index (g/m^2^)	62.77 ± 12.95	58.73 ± 13.15	0.143	58.73 ± 13.15	68.60 ± 12.78	0.000	68.60 ± 12.78	62.77 ± 12.95	0.143

*LV, left ventricle; EF, ejection fraction; E, passive filling of the LV; A, atrial filling; E', movement of the LV wall through tissue doppler; IVRT, isovolumic relaxation time; TAPSE, tricuspid annular plane systolic excursion; BNP, brain natriuretic peptide. All values are expressed as mean ± SD*.

#### Size

The systolic and diastolic diameters of LV were significantly lower in GHD children than in controls, although within normal values for age (Table 3 A.1 vs. A.2). After 6 months of treatment the systolic diameter of the LV was not modified while the diastolic diameter of the LV increased (Table 3 B.1 vs. B.2). After 6 months with recombinant GH, values of diastolic LV diameter were similar to the controls (*p* = 0.208).

#### Function

Function related parameters EF, E/A relation, E/E′ relation, and IVRT were similar in GHD children to those in controls (Table 3 A.1 vs. A.2). After treatment, we compared the GH group to the control group and found that EF and IVRT experienced slightly significant decreases, both beneficial to patients (Table 3 C.1 vs. C.2). In neither case were there any significant differences in TAPSE and BNP protein levels.

#### Mass

GH treatment resulted in a significant increase in the indexed LV mass but when we compared with control group had a value similar to the value of the basal determination of control children (Table 3 C.1 vs. C.2).

### Correlations between echocardiographic measures and growth factors

**Figure 2:**
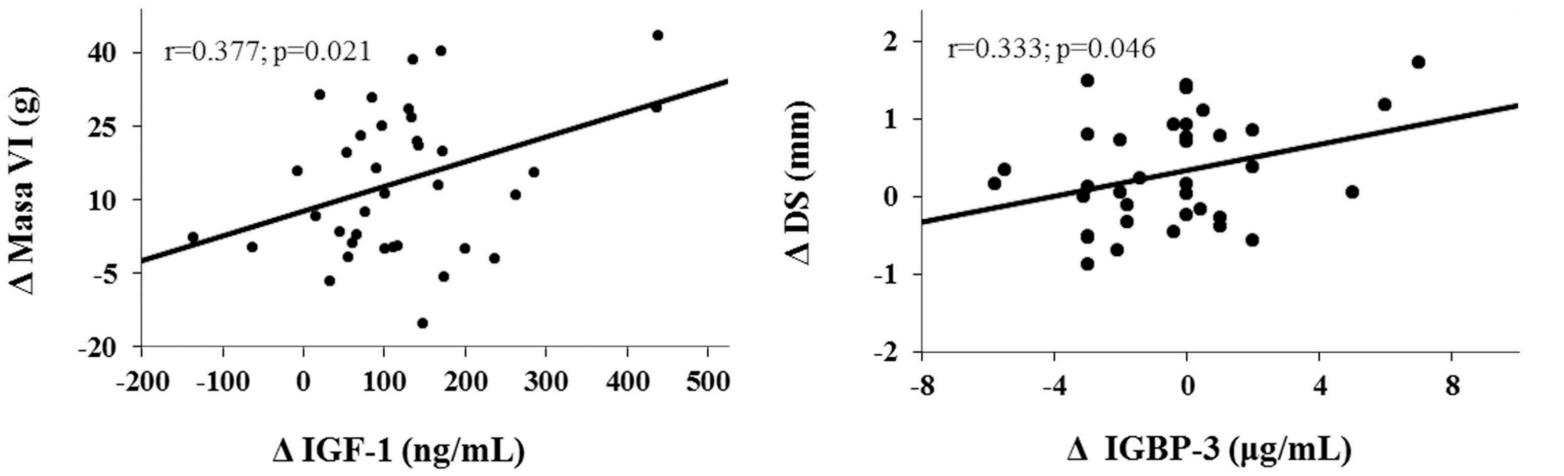
Simple linear correlation between variations in growth factor and echocardiographic measures. Changes in LVM and IGF1 levels and changes in sD and IGFBP3 levels are expressed as the difference between values after 6 months of treatment and baseline values (Δ).

Changes (Δ) in all these variables were expressed as the difference between GHD values at baseline and after treatment (Figure 2). After 6 months of treatment, a significant positive correlation was observed between changes in sD and changes in IGFBP3 levels. Correlations were also noted between changes in LVM and changes in IGF-1 levels.

## Discussion

In view of the consequences of GHD in adults, it is interesting to evaluate the cardiovascular risk in children with deficiencies and the benefit of GH replacement therapy at an early age. The study focused only on prepubertal children because the action of GH on the heart and the effects of short-term replacement therapy are less well-known (2). In this study, we compared the biochemical and cardiological profiles of children with GHD vs. healthy children and children with GHD after 6 months of treatment to provide information and/or assess whether or not this therapy is useful for preventing future complications.

Adult subjects with GHD have elevated insulin levels at baseline, and increased insulin resistance (13–15). Alterations in hydrocarbon metabolism presented by GHD patients are partly related to the changes in body composition associated with this disorder. Therapy with GH seems to act on the hydrocarbon metabolism by increasing insulin levels and resistance markers for this hormone (16, 17).

Glucose, insulin and HOMA-IR levels increased, and the QUICKI index was equal by the sixth month of treatment to controls due to GH's diabetogenic effect. The HOMA-β index showed no alterations. Children with GHD may increase their insulin resistance after treatment, mainly during the first year, and experience transient disorders in glucose metabolism. IGF-1 may be related to the control of insulin sensitivity and may be of great value in the GH-insulin equilibrium (18–20).

In adult subjects, untreated GH deficiency is associated with lipid disorders (with an increase in total cholesterol, LDLc and triglycerides, and a decrease in HDLc), as well as with alterations in glucose metabolism, coagulation, fibrinolysis and obesity, thus creating a greater risk of cardiovascular disease (21, 22).

Most authors coincide in indicating that, in the short-term, GH therapy improves LDLc and total cholesterol levels in adults (23).

Improvements in lipid metabolism are also described for GHD children being treated (24).

As in previous studies, our results confirm that GHD patients show an unfavorable lipid profile, which improves after treatment with GH. This study was only carried out during a space of 6 months, but it has recently been noted in SGA children treated with GH that the improvement in the lipid profile continues with no negative effects on glucose metabolism, although resistance to insulin is still present (18–20).

This study presents similar results to those described by other authors, finding high levels of total cholesterol and LDLc in GHD children compared to healthy children. Such levels improved after 6 months of treatment, with decreased LDLc and increased HDLc values. It shall be noted that only prepubertal children were included in the study, so we can indicate that these disorders appear at a very early age in GHD children, and they benefit from replacement therapy with GH.

The GH pleiotropic effect is well-known. Its main action is promoting linear bone growth, but it has other effects, for example, on the heart. The GH/IGF-I axis increases heart contractility, regulates heart growth and influences the cardiovascular system. GH acts on IGF-1 synthesis and release, since it has been shown that there are receptors in cardiomyocytes for GH and IGF-1; the latter will cause hypertrophy and delays in the apoptosis of these cells. It cannot be assumed that all the GH action on the heart is caused only by GH, but by the GH/IGF-1 axis (25–28).

Our results indicate that GH deficiency has effects on the heart, leads to a smaller heart size, and LVM with normal function. There were no cases of LV hypertrophy. The fact that controls did not receive follow-ups is one study limitation (since we cannot know whether or not they manifested some degree of indexed LVM increase). Nonetheless, in our study, after replacement therapy, heart size and LVM increased, with no negative effects on diastolic function. There are studies establishing similar changes to those observed in this study but in a shorter time period, not < 12 months (10, 29–32). Treatment with this hormone has short-term effects, which do not seem to have been previously described. And it also coincides with the period of a greater increase in growth rate.

GH appears to be effective and safe in heart-transplanted children who suffer GHD. After the transplant, replacement therapy with GH improved heart size, as well as function and LVM (33).

BNP is a peptide extensively used as a marker for left heart function (34). Adults with GH deficiency have shown high BNP levels that do not decrease after short-term treatment (8), although knowledge about BNP use in GHD children is limited. This study introduced the determination of BNP in GHD children in order to assess heart function at baseline and after 6 months of GH treatment. There were no alterations in their levels compared to the controls, thus suggesting that there is no ventricular dysfunction in prepubertal children with GHD.

Different correlations were established between IGF-1 and IGFBP3 with different cardiologic variables, showing significant results in LVM-IGF-1 and sD-IGFBP3. The increase in LVM could be caused both by the IGF-1 and by free GH, since cardiomyocytes have receptors for both GH and IGF-1 (35).

In summary, GHD in childhood is associated with a smaller LVM, with preserved heart function. Replacement therapy with GH increases ventricular mass, maintaining both systolic and diastolic normal functions. Furthermore, alterations in lipid metabolism associated with GHD are present in children with this deficiency from very early ages, and replacement therapy significantly improves their lipid parameters. GHD children show, from an early age, changes in size and in the indexed LV mass, similar to the changes described in adults without heart dysfunction or BNP alterations. Therapy with GH seems to have a beneficial effect on the heart.

## Author contributions

EG-G is responsible for all data collection and samples of all participants at the Hospital Reina Sofía (Córdoba). She wrote the manuscript and approved the final manuscript as presented. MC designed the study, performed the data, and took samples from all participants at the Hospital Reina Sofía (Córdoba), and approved the final manuscript as presented. LJ-R collaborated in data analysis, interpretation and discussion, and approved the final manuscript as presented. MV collaborated in data analysis, interpretation and discussion, and approved the final manuscript as presented. RC is responsible and coordinator for recruiting children at the Hospital Reina Sofía (Córdoba) and approved the final manuscript as presented. JC-V created the sampling and supervised data collection. He participated in the interpretation and discussion of the data, reviewed and edited the manuscript, and approved the final manuscript as presented. All authors have read and approved the final manuscript and assume full responsibility for its contents.

### Conflict of interest statement

The authors declare that the research was conducted in the absence of any commercial or financial relationships that could be construed as a potential conflict of interest.
